# JAK2/STAT3 Inhibition Attenuates Noise-Induced Hearing Loss

**DOI:** 10.1371/journal.pone.0108276

**Published:** 2014-10-02

**Authors:** Teresa Wilson, Irina Omelchenko, Sarah Foster, Yuan Zhang, Xiaorui Shi, Alfred L. Nuttall

**Affiliations:** 1 Oregon Hearing Research Center, Oregon Health & Science University, Portland, Oregon, United States of America; 2 Virginia Merrill Bloedel Hearing Research Center, University of Washington, Seattle, Washington, United States of America; 3 Kresge Hearing Research Institute, The University of Michigan, Ann Arbor, Michigan, United States of America; Universitat Pompeu Fabra, Spain

## Abstract

Signal transducers and activators of transcription 3 (STAT3) is a stress responsive transcription factor that plays a key role in oxidative stress-mediated tissue injury. As reactive oxygen species (ROS) are a known source of damage to tissues of the inner ear following loud sound exposure, we examined the role of the Janus kinase 2 (JAK2)/STAT3 signaling pathway in noise induce hearing loss using the pathway specific inhibitor, JSI-124. Mice were exposed to a moderately damaging level of loud sound revealing the phosphorylation of STAT3 tyrosine 705 residues and nuclear localization in many cell types in the inner ear including the marginal cells of the stria vascularis, type II, III, and IV fibrocytes, spiral ganglion cells, and in the inner hair cells. Treatment of the mice with the JAK2/STAT3 inhibitor before noise exposure reduced levels of phosphorylated STAT3 Y705. We performed auditory brain stem response and distortion product otoacoustic emission measurements and found increased recovery of hearing sensitivity at two weeks after noise exposure with JAK2/STAT3 inhibition. Performance of cytocochleograms revealed improved outer hair cell survival in JSI-124 treated mice relative to control. Finally, JAK2/STAT3 inhibition reduced levels of ROS detected in outer hair cells at two hours post noise exposure. Together, these findings demonstrate that inhibiting the JAK2/STAT3 signaling pathway is protective against noise-induced cochlear tissue damage and loss of hearing sensitivity.

## Introduction

The generation of reactive oxygen species (ROS) is one of the underlying causes of noise-induced damage to tissues in the inner ear [1]–[5]. The exact mechanisms that initiate this process are not well understood, but are thought to be due in part to ischemia/reperfusion injury as well as metabolic overstimulation [2], [4], [6]–[8]. The cellular response to ROS-induced tissue injury in the cochlea is mediated by the actions of several oxidative stress-responsive signaling pathways including nuclear factor NF-kappa-B (NF-κB), p38 mitogen-activated protein kinase, and c-Jun-N-terminal kinase (JNK) [1], [9], [10]. STAT3, part of Janus kinase/signal transducer and activator of transcription (JAK/STAT) signaling pathway, is a mechanism for transducing extra-cellular signals into a transcriptional response. Cell receptor binding by cytokines and growth factors including interleukin-6 (IL-6), IL-11, epidermal growth factor, and vascular endothelial growth factor can induce STAT3 phosphorylation by JAK and other tyrosine kinases resulting in increased transcription of an array of target genes [11]–[13]. Additionally, ischemia and oxidative stress modulate STAT3 activities through oxidation-reduction (redox) mechanisms [14], [15]. Key transcriptional targets of STAT3 are involved in cell survival, proliferation and differentiation pathways. However, increasing evidence also points to an important regulatory role for the JAK2/STAT3 signaling pathway in cellular oxidative stress injury, as inhibition of JAK2/STAT3 signaling activity reduces hydrogen peroxide-induced cell death [16]–[18]. Further, a transcription-independent mechanism for mediating increased NADPH oxidase ROS production by JAK2/STAT3, potentially through protein-protein interactions, may exist [19].

The regulation of STAT3 activity is complex and occurs on many levels from the formation of heterodimers with STAT1 and STAT5 to a variety of post-translational modifications including phosphorylation, acetylation, and methylation all of which can affect cellular localization, dimerization and gene targeting [12]. Phosphorylation of STAT3 tyrosine 705 in the cytoplasm leads to dimerization and nuclear translocation where STAT3 binds to specific DNA elements and regulates transcription of target genes [11]–[13]. In addition to being capable of activating transcription alone, STAT3 can interact with other cellular stress-activated transcription factors including hypoxia inducible factor 1α, NF-κB, and redox factor 1 enhancing their transcriptional activity [20], [21].

In this study, we examined the role of the JAK2/STAT3 signaling pathway in noise-induced damage to cochlear tissues and loss of hearing sensitivity in CBA/CaJ mice. We used a moderately damaging level of loud sound exposure, and the specific inhibitor, JSI-124, to reduce JAK2/STAT3 phosphorylation and activity. The effect of JSI-124 on noise-induced expression of STAT3 target genes was examined. Then, the functional consequence of JAK2/STAT3 inhibition on hearing sensitivity and outer hair cell (OHC) survival was determined. Finally, the role of JAK2/STAT3 in noise-induced ROS production in OHCs was assessed.

## Methods

### Animals

Male CBA/CaJ mice (The Jackson Laboratories) aged 9 to 10 weeks with normal hearing were used. All experiments were conducted in accordance with the recommendations in the *Guide for the Care and Use of Laboratory Animals* of the National Institutes of Health. The associated protocols were approved by the Institutional Animal Care and Use Committee of the Oregon Health & Science University (Animal Welfare Assurance #A3304-01).

### Drug Treatment

Mice were injected intraperitoneally (IP) with 1 mg/kg JSI-124 (cucurbitacin I) (Calbiochem, San Diego, CA) for either 3 consecutive days at 48 hours, 24 hours and 1 hour prior to noise exposure or at 1 hour prior to noise exposure as noted in text. The control group received an equal volume of DMSO (vehicle). All studies that included JSI-124 treatment consisted of 4 test groups: control (DMSO treated), JSI-124 treated, control plus noise exposure, and JSI-124 treated plus noise exposure.

### Acoustic Trauma

Mice were put into a small divided wire mesh cage and placed into an open field acoustic chamber. A free field broadband noise level of 0 or 110 dB/SPL, 4–48 kHz with a 5 minute ramp up in noise levels was applied for 3 hours.

#### Auditory Brainstem Response Threshold

The animals were anesthetized with xylazine (10 mg/kg, i.m., IVX; Animal Health Inc., Greeley, CO) and ketamine (40 mg/kg, i.m.; Hospira, Inc., Lake Forest, IL), and placed on a heating pad in a sound-isolated chamber. The external ear canal and tympanic membrane were inspected using an operating microscope to ensure the ear canal was free of wax and that there was no canal deformity, no inflammation of the tympanic membrane, and no effusion in the middle ear. Needle electrodes were placed subcutaneously near the test ear, at the vertex and at the shoulder of the ‘test ear side”. Each ear was stimulated separately with a closed tube sound delivery system sealed into the ear canal. The auditory brain-stem response to a 1-ms rise-time tone burst at 4, 8, 12, 16, 24, and 32 kHz was recorded and thresholds obtained for each ear. The intensity of tone burst stimulus was increased in steps of 5 dB. Threshold was defined as an evoked response of 0.2 µV from the electrodes. ABR measurements were taken prior to (baseline), at 1 hour post (temporary threshold shift), and at 2 weeks (permanent threshold shift) post noise exposure.

#### Distortion Product Otoacoustic Emissions

The distortion product otoacoustic emissions (DPOAE) stimuli consisted of two primary tones (f2:f1 = 1.2) at the level (L1 = L2) 60 dB SPL, were emitted and presented over a range 4–32 kHz. The sound stimuli were generated by 24 bit 192 kHz ESI Wave terminal 192X Sound Card and an in house developed acoustic system. The DPOAE stimuli were delivered to the ear canal using a coupler tip fitted within the opening of the ear canal to form a closed acoustic system. On the graphs, the amplitude of the 2f1-f2 distortion product was plotted against the f2 frequency where the DP is generated. The measurements were taken prior to (baseline) and at 2 weeks post noise exposure (PTS).

### Immunochemistry

Four hours post noise exposure, the mice were deeply anesthetized with ketamine hydrochloride (100 mg/kg, i.m.) and xylazine hydrochloride (10 mg/kg) and killed by decapitation. The cochleae were rapidly dissected from the animals and perfused with 4% paraformaldehyde in 0.1M PBS and rotated over night at 4°C. For immunohistochemistry of paraffin sections, two groups of 3 mice each were used: control non-noise exposed and noise exposed. Following decalcification in 10% EDTA for 96 hours, cochleae were embedded in paraffin, cut into 5 µm sections and placed on microscope slides. The slides were dewaxed in citrisolve and hydrated through an alcohol gradient. Heat mediated antigen retrieval was then performed in Tris/EDTA buffer (pH 9.0) and the tissue sections were blocked in 5% normal goat serum/TBS+Tween-20 (NGST) for 1 hr. The specimens were incubated overnight at 4°C with rabbit anti-pSTAT3 (Y705) antibody (#9145, Cell Signaling Technology, Danvers, MA) diluted 1∶250 in NGST, washed in TBST, and then incubated with ImmPRESS anti-rabbit Ig reagent (Vector Laboratories, Burlingame, CA) for 30 min at room temperature. The primary antibody was visualized by incubation with diaminobenzidine (Sigma Aldrich, St. Louis, MO). Tissues were visualized on a Leica DM LB light microscope (Buffalo Grove, IL) fitted with a Leica DFC295 digital microscope color camera. For fluorescent labeling of STAT3α and pSTAT3 in the organ of Corti, two groups of 3 mice each were used: control non-noise exposed and noise exposed. Following decalcification, the cochlea were embedded in 4% agarose and 50 µm sections were cut on a Leica VT1000 vibratome as described [22]. The tissue sections were immunolabeled with rabbit anti-STAT3α (1∶100, #8768, Cell Signaling Technology, Danvers, MA) or anti-pSTAT3 (Y705) antibody (#9145) at 1∶100 dilution overnight at 4°C followed by incubation with donkey anti-rabbit secondary antibody (Alexafluor 488, Life Technologies, Grand Island, NY). Nuclei were stained with Hoescht 33258 (Sigma Aldrich). The fluorescent signal was visualized on an Olympus IX81 inverted microscope fitted with an Olympus Fluoview FV1000 confocal laser microscope system (Center Valley, PA).

### Immunoblot Analysis

Whole cochleae were homogenized with a glass pestle in ice-cold lysis buffer (T-PER buffer, Thermo Scientific, Waltham, MA) containing protease and phosphatase inhibitors (HALT, Thermo Scientific). One mouse was used per sample (2 cochleae), n = 3 mice per group. After a 20 min incubation on ice, the lysate was centrifuged at 14 000× g at 4°C for 20 minutes. Protein concentration was measured using the Bio-Rad Protein Assay BCA kit. Twenty µg total protein of each sample was separated by SDS-PAGE and transferred to a PVDF membrane. The blots were blocked with 5% non-fat dry milk in TBST and incubated overnight at 4°C with primary antibodies against pSTAT3 (Y705) (#9145), total STAT3 (#4904, Cell Signaling Technology) or GAPDH (#2118, Cell Signaling Technology) diluted in 5% BSA/TBST. After washing with PBS, the membranes were incubated for 1 hr with peroxidase-conjugated goat anti-rabbit second antibody (Cell Signaling Technologies) and visualized using SuperSignal West Femto reagent (Thermo Scientific).

### Gene expression analysis by quantitative RT-PCR

Four groups of 3 mice each were used for this study: control (DMSO treated), JSI-124 treated, control plus noise exposure, and JSI-124 treated plus noise exposure. Bilateral cochleae were used as a single sample. Four hours post noise exposure, the mice were deeply anesthetized with ketamine hydrochloride and xylazine hydrochloride and killed by decapitation. Whole cochleae from each group were quickly dissected in RNAlater (Life Technologies) and total RNA was isolated using the RNeasy Mini kit (Quiagen, Valencia, CA) following manufacturer's instructions. First strand synthesis was performed using 500 ng total RNA and the First Strand Kit (Quiagen) per instructions of manufacturer. Next, quantitative real-time PCR was performed on an ABI Step One Plus system using RT^2^ real-time SYBR Green/ROX PCR master mix (Quiagen) and predesigned RT^2^ qPCR Primers for mouse IL-6, MCP1, NOX3 and VEGF (Quiagen). Thermal cycle conditions were set as: 95°C for 10 min, then 40 cycles: 95°C for 15 sec and 60°C for 1 min followed by a melt curve. Mouse *GAPDH* expression was used as an endogenous control.

### Hair cell counts

Two weeks post noise exposure after ABR and DPOAE measurements, each mouse cochlea was perfused and incubated (1 hour at room temperature) with succinic dehydrogenase (SDH) staining solution and fixed overnight in 10% formalin. After cochlear bony wall removal, the cochlear coils were carefully separated, and the lateral wall was cut off. Flat pieces of the organ of Corti were mounted on microscope slides for a light microscope cell counting procedure. A cytocochleogram was prepared for each ear. The number of present and absent hair cells was assessed throughout the entire length of the organ of Corti by evaluating SDH staining of outer hair cells. Only cells which retained normal morphology and SDH stain were counted. The length of each cochlea was converted into frequency, as previously described [23]. For plotting purposes, data from each cochlea were grouped (chosen frequency regions ±0.2 kHz) and averaged.

### ROS assessment in organ of Corti

Four groups of 3 mice each were used for this study: control (DMSO treated), JSI-124 treated, control plus noise exposure, and JSI-124 treated plus noise exposure. Mice were injected with JSI-124 or DMSO (Control) at 1 hour prior to noise exposure. At 2 hours post noise exposure, the cochleae were harvested, the stapes removed, oval and round windows punctured and a hole made in the apex. The cochlea were then perfused with CellROX Green Reagent (5 µM, Life Technologies) and incubated at 37°C for 30 minutes. At the end of this incubation period, the cochlea were perfused extensively with PBS and then fixed in 4% paraformaldehyde for 2.5 hours. The cochleae were then decalcified in 10% EDTA overnight and the organ of Corti dissected from the lower basal turn. The dissected organ of Corti were incubated in 1% BSA+0.3% Triton x-100 for 20 minutes followed by labeling with phalloidin 647 (Life Technologies) and Hoescht 33258 (Sigma Aldrich) for 15 min. After three PBS washes, the tissues were mounted onto microscope slides in vectashield (Vector laboratories). Z-stack sections of equal size and depth (approximately 3 rows of OHCs at 20 OHCs per row) were obtained on an Olympus IX81 inverted microscope fitted with an Olympus Fluoview FV1000 confocal laser microscope system (Center Valley, PA). The fluorescent intensity for each sample was assessed using ImageJ software (v1.48, http://imagej.nih.gov/ij).

### Statistical analysis

Statistical analysis was performed using SigmaPlot (v. 12.5, Systat Software Inc., San Jose, CA). The numerical results are presented as mean ± SEM. Comparison between data sets were performed with one-way ANOVA followed by Bonferroni t-test. A *P* value of <0.05 was considered significant.

## Results

### Noise exposure increases STAT3 tyrosine 705 phosphorylation

**Figure 1:**
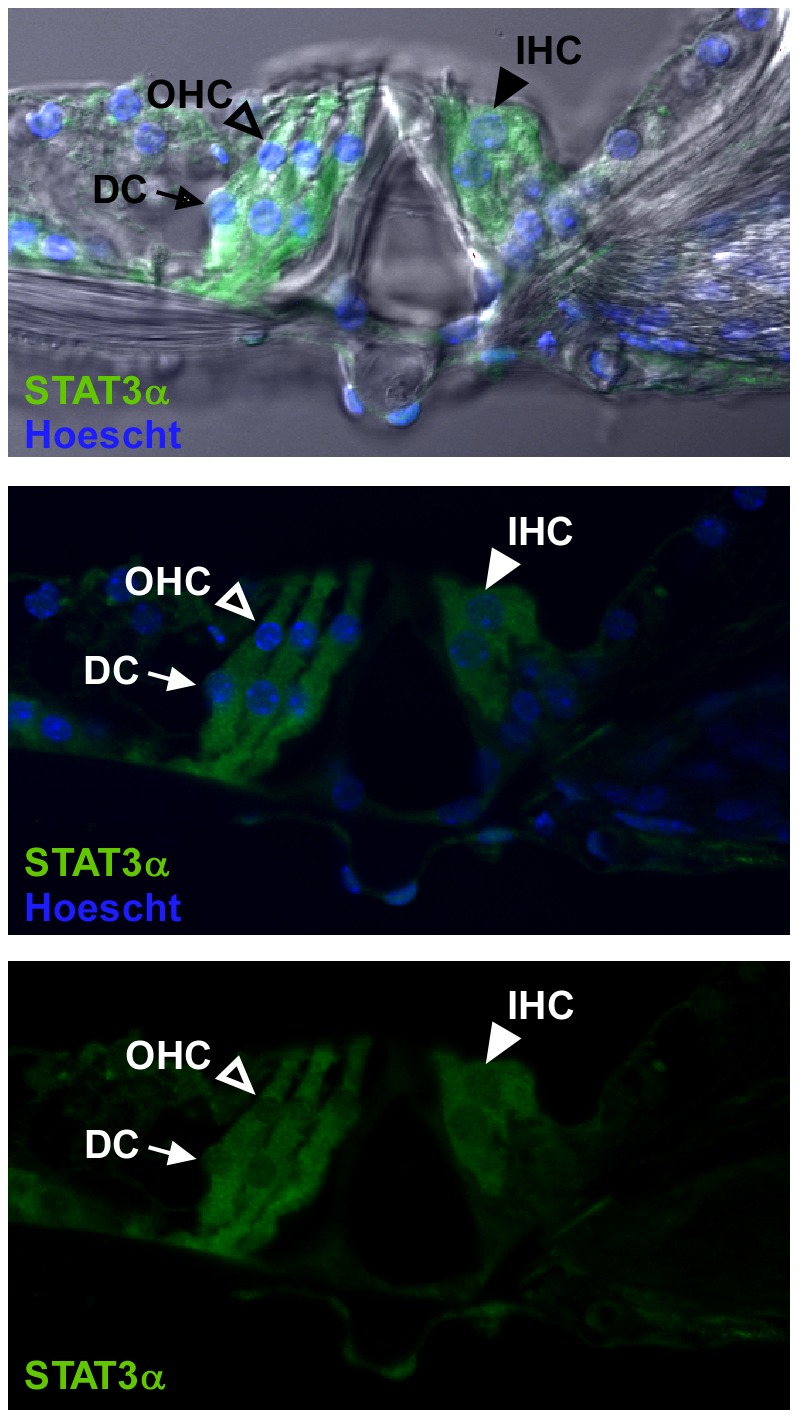
STAT3α is expressed in the sensory epithelium in the organ of Corti. Immunohistochemical staining of vibratome-cut whole mount cochlea sections for STAT3α (green) showed fluorescent signal in the cytoplasm of inner hair cells (IHC), outer hair cells (OHC) and Deiters cells (DC). Open arrow heads point to OHCs, closed arrow heads point to IHCs, and the arrows point to DCs. Nuclei are stained with Hoescht 33258 (blue). The images are from the upper basal turn of the cochlea and are representative of 3 individual mice.

**Figure 2:**
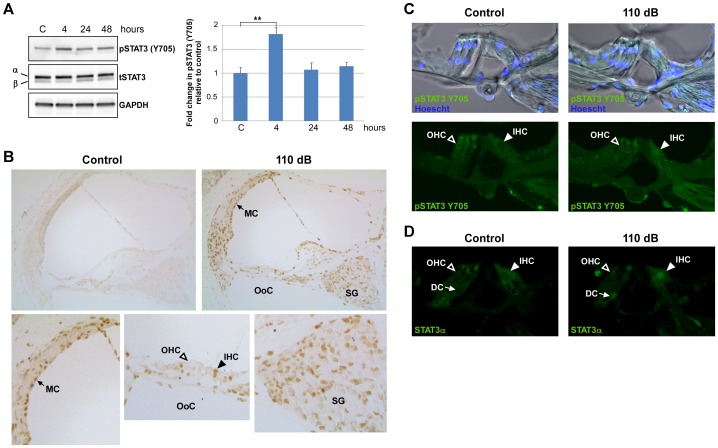
Loud exposure induces STAT3 phosphorylation in the cochlea. The effect of loud sound on the phosphorylation status of STAT3 in the whole cochlea was examined. (A) *Left Panel:* Representative Western blots of pSTAT3 (Y705) and total STAT3 (tSTAT3), both α and β isoforms, in total cochlea extracts at 0, 4, 24 and 48 hours post noise exposure (NE) (110 dB SPL, 4–48 kHz, 3 hrs). *Right Panel:* Fold change in pSTAT3 (Y705) levels relative to control at the different time points. The results were normalized to tSTAT3 levels, and GAPDH served as the loading control. Data presented as mean+SEM, n = 3 mice/time point. **P<0.001. (B) Immunohistochemical staining of paraffin sections for pSTAT3 (Y705) at 4 hours post NE. Loud sound induced STAT3 (Y705) phosphorylation was observed in many cell types including type II fibrocytes, stria vascularis marginal cells, inner hair cells and spiral ganglion cells. Enlarged images show noise-induced pSTAT3 labeling in the nuclei of marginal cells (MC, arrow), spiral ganglion cells (SG) and inner hair cells (IHC, closed arrow head), but not in outer hair cells (OHC, open arrow head) in the organ of Corti (OoC). Representative images for control and NE mice are shown, and each figure is representative of 3 individual mice analyzed for each treatment group. (C) Immunohistochemical staining of vibratome cut whole mount cochlea sections at 4 hours post NE for pSTAT3 (Y705) (green) demonstrated the presence of fluorescent signal in the IHC nuclei of noise exposed mice which was absent in the OHC nuclei. Upper panels: bright field images of pSTAT3 immunolabeled organ of Corti showing the cellular architecture of the organ of Corti. (D) Immunolabeling for STAT3α (green) at 4 hours post NE showed fluorescent signal in the IHC nuclei of noise exposed mice and not in the OHC nuclei. The immunolabeled tissue sections presented in (C) and (D) were obtained from the same treatment groups. Open arrow heads point to OHC nuclei, closed arrow heads point to IHC nuclei, and the arrows point to Deiters cell nuclei (DC). All sections (B–D) are from the mid-basal turn of the cochlea, and each figure is representative of 3 individual mice analyzed for each treatment group.

A previous study on STAT3 expression in the developing murine organ of Corti (E16 through P0) revealed that expression was primarily localized to outer hair cells (OHC) where it was proposed to be involved in OHC differentiation and survival [24]. Here, we first determined the expression pattern of STAT3 in the organ of Corti of 10 week old CBA/CaJ, mice. Immunolabeling of vibratome cut, whole mount cochlea cross-sections revealed the presence of full-length STAT3 (STAT3α) in the cytoplasm of OHCs, inner hair cells (IHCs) and Deiters cells (DC) in the organ of Corti (Figure 1). Next, to assess whether STAT3 phosphorylation is induced by noise exposure, mice were exposed to a loud sound protocol of 110 dB SPL, 4–48 kHz for 3 hours which results in a moderate amount of permanent cochlea damage and loss of hearing sensitivity. The phosphorylation status of the STAT3 tyrosine residue 705 was assessed at 4, 24 and 48 hours post noise exposure by Western-blot analysis of whole cochlea extracts (Figure 2A). At 4 hours post noise exposure, a significantly increased level of phosphorylated Y705 STAT3 (pSTAT3) (P<0.05) was present that returned to pre-exposure levels by 24 hours (Figure 2A). Levels of total STAT3 (tSTAT3) did not change following noise exposure. Both isoforms of STAT3, α and β, were present in the cochlea. The shorter STAT3β isoform possesses the Y705 phosphorylation site but lacks the C-terminal transactivation domain, both of which are present in the longer STAT3α isoform [12]. By immunohistochemical staining of paraffin sections, phosphorylated STAT3 was observed to be in the nuclei of many different cell types in the cochlea including in IHCs, stria vascularis marginal cells (MC), the spiral ganglion cells (SG), and type II, III, and IV fibrocytes at 4 hours following noise exposure (Figure 2B). Notably, while STAT3α was present in the cytoplasm of OHCs (Figure 1), pSTAT3 was not detected in the nuclei of these cells following noise exposure. We further examined whether pSTAT3 was present in OHC nuclei by fluorescent immunolabeling of vibratome-cut whole mount cochlea cross-sections which avoids the potential issue of incomplete antigen retrieval (Figure 2C). Again, no noise-induced nuclear pSTAT3 was detected in OHCs. However, the presence of pSTAT3 was observed along the plasma membrane and in the cytoplasm in both control and noise-exposed OHCs which did not noticeably change with sound exposure. In contrast, a pSTAT3 signal was present in the nuclei and entire IHC cell body of the following sound exposure (Figure 2C). In line with these results, immunolabeling for STAT3α in control and noise exposed cochlea also showed strong labeling of IHC nuclei but not OHC nuclei following noise exposure (Figure 2D). The recognition of STAT3α by the anti-STAT3α antibody is not affected by the phosphorylation status of STAT3. To examine the possibility that the presence of pSTAT3 in OHC nuclei was very transient following noise exposure, cochlea were harvested immediately after noise exposure and subjected to immunoanalysis. Consistent with the results obtained at 4 hours post NE, pSTAT3 was detected in IHC nuclei but not in OHC nuclei immediately following NE (data not shown).

### JAK2/STAT3 pathway inhibition attenuates noise exposure-induced STAT3 phosphorylation and expression of STAT3 target genes

**Figure 3:**
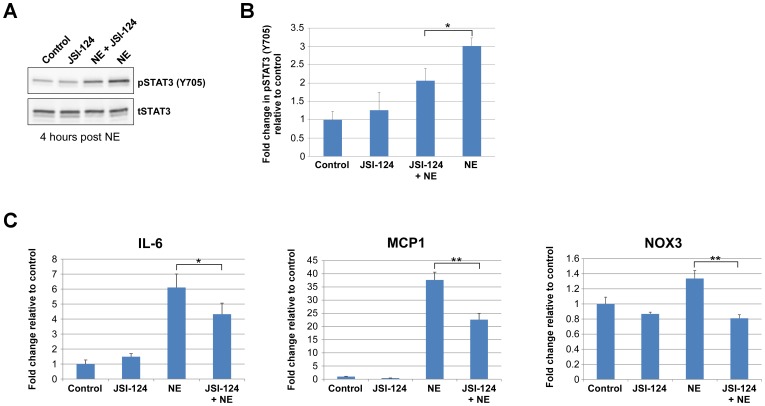
JAK2/STAT3 is involved in the loud sound induced up-regulation of pro-inflammatory transcript levels. Mice were treated with the JAK2/STAT3 pathway inhibitor JSI-124 (Cucurbitacin I) (1 mg/kg) or DMSO (Control) at 48 hours, 24 hours and 1 hour prior to exposure to 110 dB SPL 4–48 kHz for 3 hrs. (A) Representative Western blots of total cochlea extracts for levels of pSTAT3 (Y705) and total STAT3 at 4 hours post noise exposure (NE) in four different treatment groups: Control, JSI-124, JSI-124+NE, and NE animals. (B) Fold change in pSTAT3 levels in the different treatment groups relative to Control. The noise-induced increase of pSTAT3 levels was attenuated by prior treatment with JSI-124. (C) Quantitative real-time PCR was used to assess IL-6, MCP1, and NOX3 transcript levels in total RNA extracted from whole cochlea from the four different treatment groups at 4 hours post NE. With this NE protocol, increases in all three transcript levels were observed that was attenuated by prior JSI-124 treatment. Data presented as mean+SEM, n = 3 mice/group. *P<0.05; **P<0.001.

Many proinflammatory factors are transcriptionally up-regulated in the cochlea following noise exposure, including the STAT3 target genes interleukin 6 (IL-6), monocyte chemotactic protein 1 (MCP1), and several NADPH oxidases [25]–[27]. As phosphorylation of Y705 prolongs the presence of STAT3 in the nucleus and increases its transcriptional activity, we next examined the contribution of noise-induced STAT3 phosphorylation to the expression of STAT3 target genes in the cochlea. Mice were treated with the JAK2/STAT3 pathway inhibitor JSI-124 (1 mg/kg) or an equal volume of DMSO (vehicle) at 48, 24 and 1 hour prior to exposure to 110 dB SPL 4–48 kHz for 3 hours. We utilized this multiple JSI-124 treatment protocol to reduce basal levels of phosphorylated STAT3 prior to noise exposure. At four hours post noise exposure, total cochlea extracts were analyzed by Western blot for levels of pSTAT3 and total STAT3 (tSTAT3) in control, JSI-124, JSI-124+NE, and NE treated animals (n = 3 mice/group) (Figure 3A). As in Figure 2, pSTAT3 levels were significantly increased at 4 hours following noise exposure (*P*<0.001) (Figure 2B). This noise-induced increase in pSTAT3 levels was attenuated by prior treatment with JSI-124 (*P*<0.05) (Figure 3A). To determine whether inhibition of the JAK2/STAT3 pathway would affect the transcription of STAT3 target genes, quantitative real-time PCR was performed on total RNA extracted from whole cochlea at 4 hours post noise exposure (n = 3 mice/group). The STAT3 target genes IL-6 and MCP1 were assessed [28], [29]. NADPH oxidase 3 (NOX3), which is primarily expressed in the inner ear, was also examined. The results revealed large increases in IL-6 and MCP1 transcript levels (*P*<0.001) and a modest but statistically significant (*P*<0.05) increase in NOX3 levels with sound exposure (Figure 3C). Prior treatment with JSI-124 provided a significant reduction of all three transcript levels relative to vehicle treated-noise exposed samples (IL-6: *P*<0.05; MCP1: *P*<0.001; NOX3: *P*<0.001). Transcript levels of SOD2, NOX1 and NOX4 were also assayed but not found to be significantly altered at 4 hours post noise exposure (data not shown).

### Inhibition of the JAK2/STAT3 pathway improves recovery from loud sound exposure

**Figure 4:**
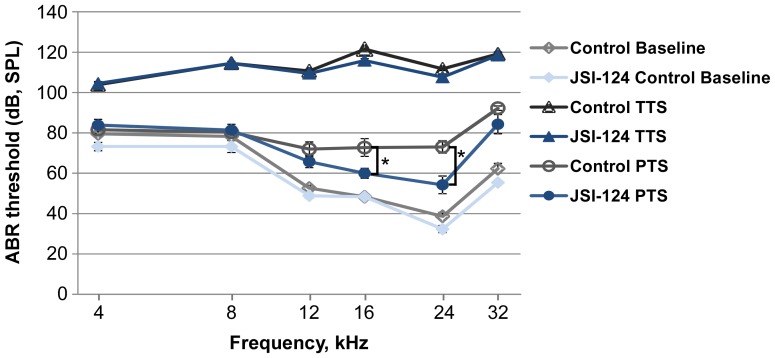
ABR threshold shifts. Inhibition of the JAK2/STAT3 pathway improves auditory brainstem response (ABR) threshold recovery from exposure to 4–48 kHz loud sound at 110 dB SPL for 3 hours. Mice were treated with JSI-124 (Cucurbitacin I) (1 mg/kg) or DMSO (Control) at 48 hours, 24 hours and 1 hour prior to sound exposure (n = 6 mice/group). ABR measurements were performed before (Baseline: normal hearing threshold), at 1 hour post (TTS: temporary threshold shift) and at 2 weeks post (PTS: permanent threshold shift) noise exposure. Significant protection was observed at 16 and 24 kHz. Data are presented as mean±SEM. *P<0.05.

**Figure 5:**
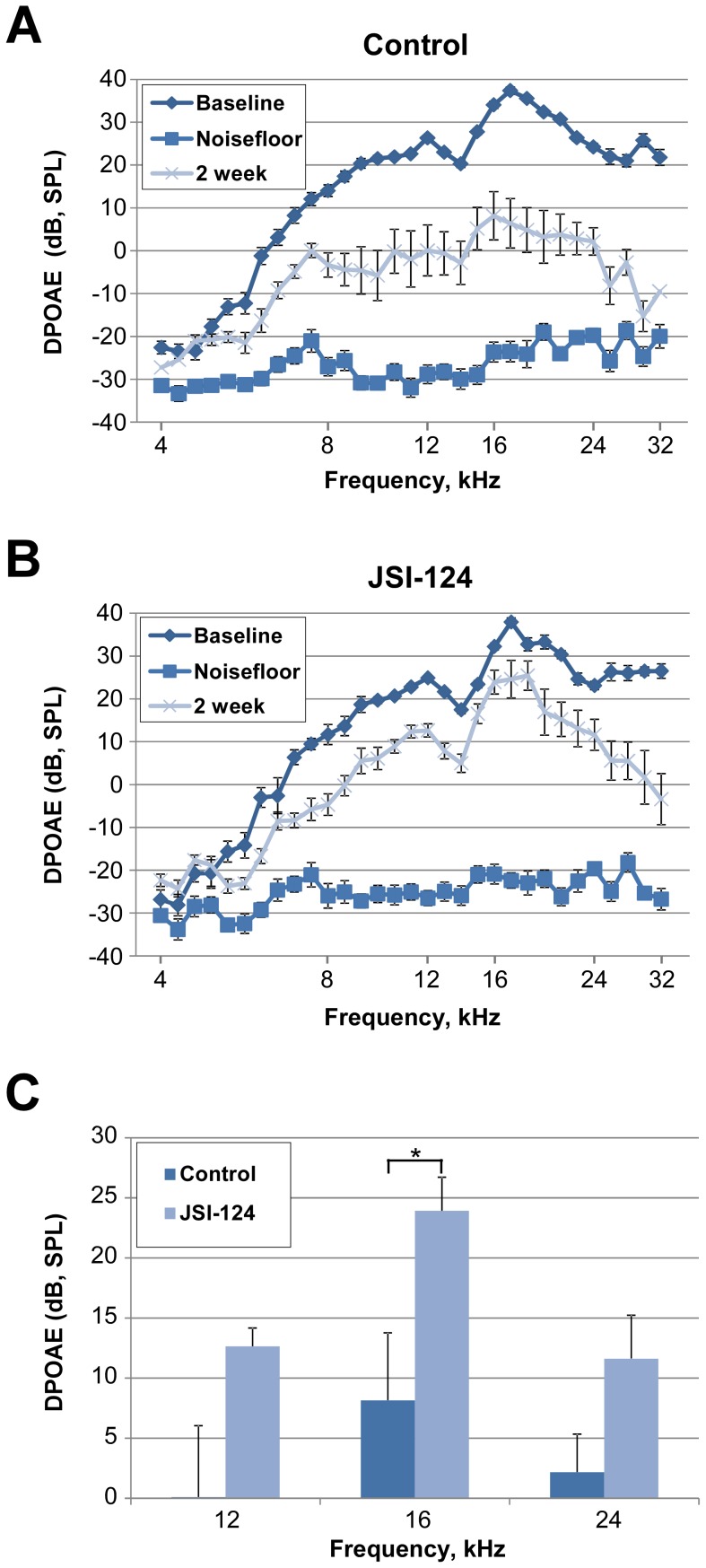
Inhibition of JAK2/STAT3 pathway improves DPOAE recovery. Inhibition of the JAK2/STAT3 pathway improves distortion product otoacoustic emissions (DPOAE) recovery from exposure to 4–48 kHz loud sound at 110 dB SPL for 3 hours. Mice were treated with JSI-124 (Cucurbitacin I) (1 mg/kg) or DMSO (Control) at 48 hours, 24 hours and 1 hour prior to sound exposure. DPOAE measurements were performed before (Baseline: normal hearing threshold) and at 2 weeks (permanent threshold shift) post noise exposure. The noisefloor is the measure of the signal created from the sum of all the noise sources and unwanted signals generated within a data acquisition and signal processing system. (A) Control group (DMSO). (B) JSI-124 treated group. Data are presented as mean±SEM, n = 6 mice/group. (C) Bar graph representation of the 12, 16 and 24 kHz data obtained at 2 weeks post noise exposure showing a significant reduction in threshold shift with JSI-124 treatment at 16 kHz. Data presented as mean+SEM. *P<0.05.

**Figure 6:**
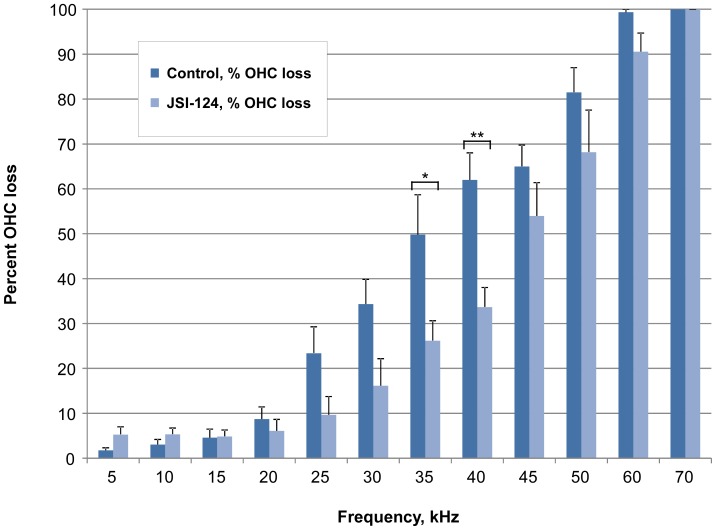
Reduced outer hair cell loss with JAK2/STAT3 inhibition. Quantification of outer hair cell (OHC) loss was performed at 2 weeks post noise exposure after auditory brainstem response and distortion product otoacoustic emissions measurements. OHCs were counted along the entire length of the cochlear sensory epithelium revealing significant reductions in OHC loss at the 35 and 40 kHz regions with JSI-124 treatment relative to control. Data are presented as mean+SEM, n = 12 cochlea/group. *P<0.05; **P<0.001.

To determine the functional consequence of JAK2/STAT3 inhibition on auditory threshold shifts following noise exposure, we measured auditory brainstem responses (ABRs, a metric of total cochlear sensitivity to sound stimulation) and distortion product otoacoustic emissions (DPOAEs, a metric of OHC function) in groups of 6 mice (12 ears) which underwent the same JSI-124 treatment protocol as described above. The sound protocol used in this study produces a moderate level of permanent threshold shift (PTS) of the ABR in CBA/CaJ mice. The ABR measurements were performed prior to (to determine normal hearing threshold: baseline), 1 hour post (to determine temporary threshold shift, TTS), and at 2 weeks post (to determine PTS) noise exposure. At 2 weeks post exposure, ABR analysis revealed that the JSI-124 treated group had significantly better threshold recovery than the control group at 16 kHz and 24 kHz (approximately 13 dB SPL (at 16 kHz) and 19 dB SPL (at 24 kHz) relative to control, *P*<0.05) (Figure 4). Similarly, DPOAE results demonstrated improved recovery of the otoacoustic emission produced by our standard sound stimulus protocol at 2 weeks following noise exposure with JSI-124 treatment relative to control (Figure 5A, B). In particular, at 16 kHz, a significant threshold reduction of approximately 20 dB SPL was observed in the JSI-124 treatment compared to control (*P*<0.05) (Figure 5C). Consistent with the ABR and DPOAE functional measurements, auditory hair cell counts revealed improved OHC survival within the basal region of the cochlea (35 kHz and 40 kHz, *P*<0.05 and *P*<0.001, respectively) for the JSI-124 treated group relative to the control group (Figure 6).

### OHC ROS production is reduced by JAK2/STAT3 inhibition

**Figure 7:**
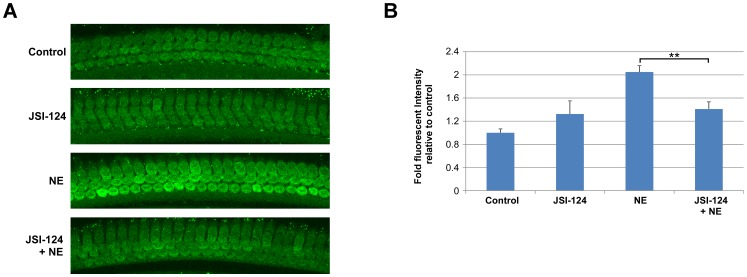
Reduced outer hair cell reactive oxygen species levels with JAK2/STAT3 inhibition. Mice were given a single injection of JSI-124 (1 mg/kg) at 1 hour prior to noise exposure (NE). (A) Representative images of surface preparations of the four different treatment groups (Control, JSI-124, NE, and JSI-124+NE) showed increased fluorescent intensity (CellROX Green, 488) at 2 hours following NE. Pre-treatment with JSI-124 reduced reactive oxygen species (ROS) levels relative to NE alone. Each of the images was taken from the mid-basal turn and each figure is representative of 3 individual mice analyzed for each treatment group. (B) Quantification of CellROX fluorescent intensity demonstrated a significant decrease in ROS levels in the JSI-124+NE group relative to the NE group. Data presented as mean+SEM, n = 6 cochlea/group. *P<0.05.

Loud sound exposure increases ROS generation in OHCs, likely from both mitochondrial and NADPH oxidase activities, which can lead to cellular damage and OHC loss. In this study, we did not detect nuclear localized STAT3α or pSTAT3 in OHCs following noise exposure (Figure 2B–D) suggesting that STAT3 is not directly involved in the increased expression of the NADPH oxidases in OHCs following noise exposure [25], [30]. However, increased NADPH oxidase activity can be stimulated in a transcription-independent manner [15], [19], [31]. Here, while no noticeable change in phosphorylation status was observed following noise exposure, pSTAT3 was present in the cytoplasm and along the plasma membrane of OHCs (Figure 2C). Therefore, we examined whether the JAK2/STAT3 signaling pathway plays a transcription-independent role in the increased ROS production in OHCs that occurs following loud sound exposure. Mice were pretreated with a single dose of JSI-124 (1 mg/kg) at one hour before noise exposure in order to inhibit noise-induced JAK2/STAT3 activity. At 2 hours post noise exposure, total ROS levels in OHCs were assessed in the mid-basal turn of the cochlea using CellROX Green reagent. The results showed a significant increase in ROS levels in OHCs from the noise exposed group relative to OHCs from non-exposed controls (2.05 fold-increase, *P*<0.001) (Figure 7A, B). Treatment with JSI-124 significantly reduced total ROS levels in the OHCs from noise exposed mice relative to non-treated noise exposed animals (*P*<0.05). The mean fluorescence intensity for each of the test groups is presented as fold-increase in fluorescent intensity over the non-noise exposed control group (n = 6 cochlea/group) (Figure 7B).

## Discussion

In this study, we show that STAT3 was activated in many cell types of the cochlea, as assessed by phosphorylation of tyrosine residue 705 and nuclear translocation, after exposure to a moderately damaging noise exposure protocol. Concomitant with STAT3 activation, up-regulated expression of the STAT3 target genes IL-6, MCP1 and NOX3 was observed. The role of the JAK2/STAT3 signaling pathway in the cochlea was examined through the use of the specific inhibitor JSI-124. Consistent with studies showing that the JAK2/STAT3 pathway is directly involved in regulating the oxidative stress response [16], [32], [33], here we found that inhibition of the JAK2/STAT3 pathway lead to improved recovery of hearing sensitivity following noise exposure with increased OHC survival and a reduction in ROS levels in OHCs.

### Cell-type specific STAT3 phosphorylation in the cochlea

STAT3 Y705 can be phosphorylated by a variety of tyrosine kinases including members of the Janus kinase family, sarcoma kinase (Src), and epidermal growth factor kinase [12], [34]. STAT3 Y705 phosphorylation increases dimerization, DNA binding and transcriptional activity prolonging its presence in the nucleus [12], [35]. However, STAT3 nuclear translocation and DNA binding can occur even in the unphosphorylated state (USTAT3), and shuttling of USTAT3 between the cytoplasm and the nucleus occurs continually enabling basal transcription levels of STAT3 target genes [36], [37]. Many STAT3 target genes are involved in the regulation of cell-survival, differentiation and cellular proliferation activities, and its functions are essential for early embryonic development [38]. The importance of STAT3 to cochlear development was first indicated by the finding of STAT3 expression being primarily in OHCs at E16 and P0, indicating an essential role in OHC differentiation and survival [24]. At 10 weeks of age, we observed STAT3α expression not only in OHCs but also in IHCs and DCs. Following noise exposure, nuclear localization of pSTAT3 was detected in numerous different cell types in the cochlea, many of which are known to undergo oxidative stress following noise exposure [2], [39], [40]. However, despite the presence of STAT3α in OHCs, pSTAT3 was not detected in the nuclei of these cells following noise exposure. Although it is certainly possible that a more severe noise exposure protocol would have resulted in pSTAT3 OHC nuclear localization, the sound level used already is sufficient to cause OHC loss.

Cell-type specific nuclear localization of pSTAT3 is reminiscent of NF-κB activation and nuclear translocation induced by loud sound exposure [41], [42] and treatment with ototoxic agents [9]. Similar to our findings with STAT3, nuclear-localized NF-κB was not observed in OHCs following noise or ototoxic drug exposure. Interestingly, co-injections of the ototoxic drug kanamycin and an antioxidant promoted the translocation of NF-κB into the nuclei of OHCs [9]. The basis for the antioxidant effect on NF-κB cellular localization may have been the change in the oxidation-reduction state in OHCs as a highly oxidant environment is present in OHCs following either noise exposure or ototoxic drug treatment. Both NF-κB and STAT3 are subject to oxidation-reduction regulation through oxidation of critical cysteine residues that reduce the DNA binding capacity and nuclear retention of these proteins [14], [43], [44]. Oxidation-reduction sensitivity may regulate the cellular localization of STAT3 as well as its interactions with other proteins and, therefore, its functional activity in a cell [12], [14]. It is possible that antioxidant treatment would also allow pSTAT3 localization to OHC nuclei altering transcriptional and physiological outcomes following noise exposure.

### Loud sound-induced STAT3 transcriptional activity in the cochlea

It is known that the JAK2/STAT3 signaling pathway plays a key role in oxidative stress-mediated tissue injury. Several STAT3 target genes, including IL-6, MCP1 and NOX1 and NOX4, are up-regulated by loud sound and are involved in the ensuing cochlear tissue damage and loss of hearing sensitivity [25], [27], [45]. To examine the role of the JAK2/STAT3 signaling pathway in the noise-induced stress response, we utilized the JAK/STAT3 inhibitor JSI-124. JSI-124, a natural plant product also referred to as cucurbitacin I, is a selective JAK/STAT3 inhibitor which rapidly suppresses the phosphorylation of STAT3 tyrosine residue 705 by the JAK kinase (complete inhibition within 1–2 hours in cell lines) and does not affect the activities of Src, Akt, extracellular-regulated kinase1/2, or JNK [46]. Here, Western blot analysis of whole cochlear extracts from mice treated with JSI-124 at 48, 24 and 1 hour prior to noise exposure revealed that this drug provided a significant reduction in the noise-induced increase in pSTAT3 levels. We next examined whether inhibiting the JAK2/STAT3 pathway would affect the transcription of several target genes and, therefore, the noise-induced stress response in the cochlea. Quantitative real-time PCR was performed on total RNA extracted from whole cochlea at 4 hours post noise exposure. For IL-6, a powerful activator of the JAK2/STAT3 pathway, noise exposure resulted in a 6-fold increase in IL-6 transcript levels which was attenuated by JSI-124 treatment. This finding is consistent with a previous study which showed that loud sound exposure lead to widespread increased expression in the lateral wall and spiral ganglion within 24 hours of exposure [26]. The IL-6 receptor IL-6R and the glycoprotein, gp130, are also present in these tissues as well as in the auditory hair cells and supporting cells in the organ of Corti [45]. The relevance of IL-6 overexpression in noise-induced hearing loss was shown through the use of an anti-IL-6 receptor antibody [45]. This study showed that IL-6 receptor blockage resulted in reduced basal levels of STAT3 Y705 phosphorylation in whole cochlea extracts and improved recovery of hearing sensitivity following noise exposure [45]. Together, these studies demonstrate a detrimental role for STAT3 in the noise-induced stress response.

Several of the NADPH oxidase isoforms, including the STAT3 target genes NOX1 and NOX4, are present in the cochlea and are involved in ROS-mediated tissue damage and loss of hearing sensitivity [25], [47]–[50]. The NADPH oxidases are expressed in a variety of different cell types such as the spiral ganglion cells, inner and outer hair cells, and fibrocytes of the lateral wall [25], [47]–[50]. Transcript levels of the NADPH oxidases and several accessory factors have been found to be altered following loud sound exposure, and noise exposure increased NOX-generated ROS in the cochlea [25], [48]. Additional studies determined that ROS-inducing cisplatin exposure increased NOX1 and NOX4 transcript and protein levels in nearly all cell types of the cochlea [49], while siRNA mediated knockdown of NOX3 in the cochlea protected against cisplatin ototoxicity [50]. Another report on NOX3 demonstrated down-regulated transcript levels at 24 hours following noise exposure [25]. However, increased NOX3 protein levels in OHCs following noise exposure have also been observed [30]. Here, we observed a modest but statistically significant (*P*<0.05) increase in NOX3 transcript levels following noise exposure which was prevented by pretreatment with JSI-124. While not statistically significant, a small decrease in NOX3 levels was detected following JSI-124 treatment alone. It is possible that basal STAT3 transcriptional activities were reduced with JSI-124 treatment accounting for this modest down-regulation. Potential reasons for the different effects of noise exposure on NOX3 expression levels between these studies include the use of different animal models and sound exposure protocols as well as the timing of NOX3 evaluation post noise exposure.

### Functional consequences of JAK2/STAT3 pathway inhibition

The sound protocol used in this study has been well-characterized by our laboratory and produces a moderate level of ABR permanent threshold shift and a characteristic cochlear morphology that includes loss of OHCs in the basal coil of the cochlea. At 2 weeks following noise exposure, a time at which organ of Corti morphology and function have stabilized, ABR and DPOAE analysis revealed that JAK2/STAT3 pathway inhibition reduced ABR threshold and DPOAE shifts. Hair cell counts corroborated the sensitivity rescue by improved survival of OHCs in the basal turn of the cochlea.

Upon noise exposure, OHC mitochondria and NADPH oxidases generate significant levels of ROS contributing to cell damage and death. The NADPH oxidases are the major source of non-mitochondrial ROS production that functions in both cellular signaling, as in the case of phagocytes, and as generator of excessive ROS in the form of superoxide that can lead to cell damage and death [51]. In addition to transcriptional up-regulation of the NADPH oxidases, increased NADPH oxidase activity can be mediated through direct protein-protein interactions. ROS production by NOX1 can be increased through direct engagement by ligand-bound tumor necrosis factor α receptor/TRADD/RIP1 complex [31]. Tyrosine phosphorylated STAT5 also increased ROS production by NADPH oxidases, likely through a direct interaction with the GTPase Rac1, a component of NADPH oxidase complexes [15]. A similar mechanism for STAT3 is suggested by the ability of STAT3 to interact with Rac1 as well [52], [53]. Further, a study by Manea, *et al.* revealed that interferon gamma (IFNγ)-induced NOX activity was reduced by JAK2 chemical inhibition, but not by STAT1 and STAT3 decoy oligodeoxynucleotides, at 4 hours post IFNγ treatment supporting a transcription-independent mechanism for STAT3 upregulation of NADPH oxidase activity [19]. Here, we observed that mice treated with a single dose of JSI-124 at 1 hour before noise exposure resulted in reduced levels of ROS in their OHCs at 2 hours post noise exposure indicating that STAT3 may be directly stimulating NADPH oxidase activity in OHCs as well.

In summary, we found that inhibiting the JAK2/STAT3 signaling pathway reduced OHC loss and improved auditory sensitivity following loud sound exposure. The protective effect of JAK2/STAT3 inhibition was likely through a combination of reduced transcriptional expression of pro-inflammatory factors and attenuated ROS production in the cochlea.
